# Assessment of the gut bacterial microbiome and metabolome of girls and women with Rett Syndrome

**DOI:** 10.1371/journal.pone.0251231

**Published:** 2021-05-06

**Authors:** Santosh Thapa, Alamelu Venkatachalam, Nabeel Khan, Mohammed Naqvi, Miriam Balderas, Jessica K. Runge, Anthony Haag, Kathleen M. Hoch, Daniel G. Glaze, Ruth Ann Luna, Kathleen J. Motil

**Affiliations:** 1 Department of Pathology, Medical Metagenomics Laboratory, Texas Children’s Microbiome Center, Texas Children’s Hospital, Houston, Texas, United States of America; 2 Department of Pathology and Immunology, Baylor College of Medicine, Houston, Texas, United States of America; 3 Department of Pediatrics, Baylor College of Medicine, Houston, Texas, United States of America; 4 Department of Pathology, Metabolomics and Proteomics Laboratory, Texas Children’s Microbiome Center, Texas Children’s Hospital, Houston, Texas, United States of America; 5 Department of Neurology, Baylor College of Medicine, Houston, Texas, United States of America; 6 USDA/ARS Children’s Nutrition Research Center, Baylor College of Medicine, Houston, Texas, United States of America; Wageningen Universiteit, NETHERLANDS

## Abstract

**Background:**

Gastrointestinal problems affect the health and quality of life of individuals with Rett syndrome (RTT) and pose a medical hardship for their caregivers. We hypothesized that the variability in the RTT phenotype contributes to the dysbiosis of the gut microbiome and metabolome in RTT, predisposing these individuals to gastrointestinal dysfunction.

**Objectives:**

We characterized the gut bacterial microbiome and metabolome in girls and young women with RTT (n = 44) and unaffected controls (n = 21), and examined the relation between the composition of the microbiome and variations in the RTT phenotype.

**Methods:**

Demographics and clinical information, including growth and anthropometric measurements, pubertal status, symptoms, clinical severity score, bowel movement, medication use, and dietary intakes were collected from the participants. Fecal samples were collected for analysis of the gut microbiome using Illumina MiSeq-based next-generation sequencing of the 16S rRNA gene followed by bioinformatics analysis of microbial composition, diversity, and community structure. Selected end-products of microbial protein metabolism were characterized by liquid chromatography-mass spectrometry.

**Results:**

The gut bacterial microbiome differed within the RTT cohort based on pubertal status (p<0.02) and clinical severity scores (p<0.02) of the individuals and the type of diet (p<0.01) consumed. Although the composition of the gut microbiome did not differ between RTT and unaffected individuals, concentrations of protein end-products of the gut bacterial metabolome, including γ-aminobutyric acid (GABA) (p<0.001), tyrosine (p<0.02), and glutamate (p<0.06), were lower in the RTT cohort. Differences in the microbiome within RTT groups, based on symptomatic anxiety, hyperventilation, abdominal distention, or changes in stool frequency and consistency, were not detected.

**Conclusions:**

Although variability in the RTT phenotype contributes to the dysbiosis of the gut microbiome, we presently cannot infer causality between gut bacterial dysbiosis and gastrointestinal dysfunction. Nevertheless, alterations in the gut metabolome may provide clues to the pathophysiology of gastrointestinal problems in RTT.

## Introduction

Rett syndrome (RTT), a neurodevelopmental disorder arising from loss of function mutations in the X-linked methyl CpG binding protein 2 (*MECP2*) gene, is a leading cause of developmental disability in children [1]. RTT affects approximately 1 in 10,000 females worldwide; *MECP2* mutations may be associated with severe neonatal encephalopathy in males [2]. The diagnosis of classic RTT is based on strict clinical criteria put forth by the Rett Syndrome Diagnostic Workgroup [3]. The clinical disorder is recognized between 6 and 18 months of age in girls who plateau in their developmental milestones and lose communication and purposeful hand skills coincident with the onset of hand stereotypies. Growth of the head slows and other neurological signs such as motor apraxia and seizures evolve [3].

Although neurological symptoms predominate, more than 90% of girls with RTT develop gastrointestinal problems that affect their health and quality of life [4–7] and pose a substantial medical challenge for their caregivers [4, 8]. Chewing and swallowing dysfunction, gastroesophageal reflux, gastroparesis, biliary tract disorders, gas bloating, and constipation complicate the clinical course of this disorder [4, 5]. Disturbances in gastrointestinal function predispose girls with RTT to individual nutrient deficits, protein-energy malnutrition, and growth failure [4, 6, 7]. The mechanisms by which *MECP2* alters gastrointestinal function in RTT is unknown, but likely to be multifactorial. Studies using a *MECP2* knock-out mouse model suggest that gastrointestinal dysmotility in RTT may arise from functional impairments in the *MECP2* gene within the enteric nervous system [9]. Strategies to treat or prevent gastrointestinal problems in RTT are limited. Unlocking the direct cause of gastrointestinal dysregulation in RTT is central to the development of treatment strategies for the gastrointestinal complications observed in individuals with RTT.

The gut microbiome is recognized as a potent driver of human health and disease [10]. Dysbiosis of the gut microbiome is associated with a number of health conditions, ranging from gastrointestinal disorders such as irritable bowel syndrome [11] to complex neurodevelopmental disorders such as autism spectrum disorders [12–15]. The gut microbiome-brain axis has been a central target to reduce or mitigate progression of various diseases associated with human gastrointestinal, respiratory, and neurological systems [16–22]. Nevertheless, reports on the role of gut microbiome in the pathophysiology of individuals with RTT are scant. Studies in two Italian cohorts of girls with RTT found altered microbiome and short- and branched-chain fatty acid profiles compared with healthy controls [23, 24]. These studies suggest that alterations in the gut microbiome and metabolome may underlie intestinal dysfunction in individuals with RTT.

In the present study, we characterized the gut bacterial microbiome and metabolome in a cohort of RTT individuals and examined the relation between the composition of the gut microbiome and variations in the RTT phenotype. We hypothesized that the variability in the RTT phenotype contributes to the dysbiosis of the gut microbiome and metabolome in RTT, thereby predisposing these individuals to gastrointestinal dysfunction.

## Materials and methods

### Study design

This study was designed as an exploratory cross-sectional, observational investigation to characterize the gut microbiome and metabolome in individuals affected with RTT and unaffected controls and to examine the relation between variations in the RTT phenotype and the gut microbiome and metabolome.

### Study participants

A total of 44 girls and young women with clinically diagnosed RTT and 21 age-matched unaffected females who served as controls were enrolled (Table 1). None of the controls had gastrointestinal disorders. None of the participants received antibiotics during the 6 months prior to sample collection.

**Table 1:** Demographic and clinical characteristics of individuals with Rett syndrome (RTT) and unaffected Controls (C).

Characteristics of subjects	Group of Individuals*		p*-*value^+^
	RTT	C	
*Demographics*			
Number of participants	44	21	-
Age (y)	12.4 (5.1, 36.1)	10.3 (4.9, 27.8)	NS
Race (W:H:B:A:O) (%)**	57:27:11:2:2	52:24:14:0:10	NS
*Pubertal Status*			
Menarche achieved (%)	50	33	NS
Pre-pubertal: Post-pubertal (%)	20:80	52:48	0.01
*MeCP2 Mutation*			
Participants (n)	43	-	-
Mutation severity^#^ (mild:moderate:severe) %	33:44:23	-	-
*Growth measurements*			
Height (z-score)	-2.5 (-5.7, 1.8)	0 (-2.5, 3.3)	0.001
Weight (z-score)	-1.9 (-10.4, 0.7)	0.4 (-3.2, 2.5)	0.001
Body mass index (z-score)	-0.5 (-8.7, 1.5)	0.4 (-3.2, 2.2)	0.01
Body fat (%)	26 (17, 37)	26 (15, 39)	NS
Triceps skinfold (mm)	11 (5, 33)	14 (5, 31)	0.04
Arm muscle area (mm^2^)	2332 (1418, 5314)	2704 (1282, 4730)	NS
*Symptoms*			
Anxiety (%)	52	14	0.01
Salivary cortisol (n)	42	20	-
Cortisol (μg/dL)	0.12 (0.03, 1.37)	0.08 (0.02, 0.23)	0.01
Bruxism (%)	68	5	0.001
Hyperventilation (%)	68	0	0.001
Abdominal distention (%)	50	0	0.001
Seizures (%)	64	0	0.001
*Bowel Movement*			
Frequency < 1/day (%)	41	25	NS
Stool consistency			0.001
Loose (diarrhea)	27 (61%)	3 (14%)	-
Normal	10 (23%)	16 (76%)	-
Formed (constipated)	7 (16%)	1 (5%)	-
Data not available	-	1 (5%)	-
*Ambulatory Status*			
Non-ambulatory (%)	39	0	0.001
*Clinical Severity Score (CSS)*			
Participants (n)	40	-	-
CSS value	25 ± 7	-	-
CSS^##^ (mild:moderate:severe) %	30:45:25	-	-
*Medications*			
Proton pump inhibitors (%)	61	0	0.001
Prokinetics (%)	18	0	0.05
Probiotics (%)	23	0	0.01
Laxatives (%)	70	0	0.001
Anticonvulsants (%)	59	0	0.001
Vagal nerve stimulation therapy (%)	16	0	0.01
*Gastrointestinal Health Questionnaire (score)*			
Health/Pain	13 (0, 43)	0 (0, 5)	0.001
Eating/Chewing/Swallowing	24 (1, 48)	0 (0, 7)	0.001
Gastrointestinal	23 (0, 52)	0 (0, 13)	0.001
Mood/Personality	10 (0, 40)	0 (0, 8)	0.001
Parental Concerns	10 (0, 51)	0 (0, 5)	0.001
Total	71 (14, 231)	1 (0, 7)	0.001

*Values expressed as median (minimum, maximum) or proportion (%).

**W = White (Caucasian), H = Hispanic, B = Black (African American), A = Asian, O = Other (Pacific Islander).

^#^*MeCP2* mutation severity: Mild = *R133C*, *R294X*, *R306C*, 3’ truncations; Moderate = *T158M*, all others except mild/severe; Severe = *R106W*, *R255X*, *R270X*, large deletions.

^##^Clinical severity score (CSS): Mild = ≤ 19, Moderate = 20–30, Severe = ≥31.

^+^ Mann-Whitney test (continuous variables) and Chi-squared test (discrete variables), p-value ≤ 0.05 considered significant.

### Ethical approval

The study was approved by the Institutional Review Board at Baylor College of Medicine (Houston, TX, USA). Written informed consent was obtained from the parents of all RTT girls and young women, as well as those of unaffected girls younger than 18 y of age. Written informed consent was obtained directly from unaffected young women older than 18 y of age. The assent of individuals with RTT and unaffected girls younger than 12 y of age was waived due to their cognitive disability or degree of maturity.

### Collection of demographic, clinical, and dietary information

Demographic information, including age, race or ethnicity, pubertal status, and age at onset of menarche, were recorded for each participant. The pre- and post-puberty classification was based on Tanner staging, i.e., breast and pubic hair changes during pubertal development. The clinical severity score (CSS) for RTT was classified as mild (CSS ≤ 19), moderate (CSS 20–30), or severe (CSS ≥ 31) based on the scale derived from the Natural History of Rett Syndrome Study [25]. The clinical severity of *MECP2* mutations were classified as mild for R133, R294, R306C, and 3’truncation mutations; moderate for T158M and all other mutations except the severe group; and severe for R106W, R255X, R270X, and large deletion mutations [26]. Growth and anthropometric measurements including height or length, weight, arm circumference, and triceps, biceps, subscapular, and suprailiac skinfold thickness measurements were obtained using standard clinical techniques. Body mass index (BMI) was calculated from height and weight measurements. Height or length, weight, and BMI measurements were converted to z-scores. Body fat, expressed as a proportion of body weight, was calculated from the sum of the four skinfold thickness measurements [27]. The clinical phenotype was characterized by age, BMI, clinical severity score, and the presence or absence of gastrointestinal symptoms including anxiety, bruxism, hyperventilation, abdominal distension, bowel movement frequency, stool consistency, and gastrointestinal health quality, as measured by the Gastrointestinal Health Questionnaire (GHQ) [28]. Dietary energy and nutrient intakes were determined from food records. Parents recorded the type, quantity, and frequency of food and beverage consumption for three days, including one weekend day, to determine daily nutrient intakes. A research dietitian estimated total daily dietary energy, macronutrient, and selected micronutrient intakes using a computer-based software application (Nutrition Data System for Research (NDSR), University of Minnesota Nutrition Coordinating Center, Minneapolis, MN, Version 2017).

### Sample collection

#### Fecal samples

Stool samples from 65 participants (one sample/individual) were collected in an OMNIgene•GUT kit (DNA Genotek Inc., Ontario, Canada) to characterize the gut bacterial microbiome. Aliquots from the collection kit were also used for biochemical analysis of specific metabolites. The samples were stored at -80°C until processing.

#### Saliva and blood samples

Saliva (0.5 ml) samples were obtained at timed intervals from each individual for the measurement of cortisol concentrations using liquid chromatography-mass spectrometry (Quest Diagnostics, Secaucus, NJ). Blood samples (5 ml) from RTT patients were collected by sterile venipuncture to measure plasma amino acid concentrations using the Biochrom Amino Acid Analyzer 30 (Harvard Bioscience, Inc., Holliston, MA).

### DNA extraction and 16S amplicon sequencing

DNA extraction, amplification, and sequencing were performed at the Medical Metagenomics Laboratory in the Texas Children’s Microbiome Center. Genomic DNA was extracted from stool samples using the MoBio PowerSoil DNA Isolation kit (Qiagen, Hilden, Germany) following manufacturer recommendations. Quantification of the resulting DNA was performed on the Qubit® 2.0 fluorometer (Thermo Fisher Scientific, Inc., Wilmington, DE) using a high-sensitivity double-stranded DNA (dsDNA) assay kit. The DNA samples were stored at -80°C until further processing.

The V4 region of the 16S rRNA gene was amplified by PCR as previously described [29]. The pooled amplicon libraries were sequenced using a 500 cycle v2 chemistry kit (2×250 bp) on the Illumina MiSeq platform following the standard Illumina sequencing protocol [30]. No template PCR controls (i.e. negative controls, n = 2) were sequenced along with the samples (n = 65) to monitor the potential background noise.

### Sequence processing

The raw reads were processed as described in Thapa et al [31]. Next, the sequences were quality filtered using the LotuS pipeline (v1.462) [32] and processed as previously described [31, 33]. Chloroplast-derived and mitochondrial sequences were removed from the biom file before proceeding with analysis, along with the OTUs that fail to classify as bacteria at the kingdom level and unclassified OTUs at the phylum level. Samples with <1000 read counts were excluded from analysis [34, 35].

### Sequence analysis

Relative abundance of bacterial taxa at various taxonomic levels were calculated for each sample using the statistical analysis of metagenomic profiles (STAMP) (v2.1.3) software [36]. Diversity analyses were performed in a dataset generated after rarefying or evenly subsampling (without replacement) the data to the lowest sequencing depth (1824 reads per sample) to overcome the potential bias of unequal sequencing depth among samples.

Observed OTUs and ACE value as measurements of bacterial richness (alpha diversity) were calculated using the ‘phyloseq’ R-package (v1.32.0) [37]. Fisher’s alpha index of diversity, a diversity index relatively unaffected by sample size variation and completely independent for samples with greater than 1000 reads [34, 35], was calculated using the ‘microbiome’ R-package (v1.10.0) [38]. Faith’s phylogenetic diversity, the alpha diversity metric which takes into account of the phylogenetic tree of OTUs contained in a sample, was calculated using the ‘picante’ R-package (v1.8.1) [39].

Principal coordinates analysis (PCoA) plots of the Bray-Curtis dissimilarity index and UniFrac distance metrics [40], as measures of beta diversity between samples, were generated using ‘phyloseq’ R-package. A phyloseq-class object containing an otu-table and otu-table plus phylogenetic tree was used as input for calculating the Bray-Curtis and UniFrac distance metrics, respectively. Additionally, principal component analysis (PCA) ordination of the Aitchison distance [41], a compositionally aware distance metrics, following the centered log-ratio (CLR) transformation of the read counts [42] was used to compare the community composition (beta diversity) between RTT and control groups. The Aitchison distance was calculated using the ‘microbiome’ package in R. The inter-individual divergence (beta diversity) with respect to the median profile within RTT and control groups was calculated using the ‘microbiome’ R-package [38].

### Mass spectrometry for targeted metabolomics

Liquid chromatography-mass spectrometry (LC-MS) analysis of the stool samples was performed using a Shimadzu (Kyoto, Japan) Nexera-XR ultra-high performance liquid chromatography (UHPLC) system coupled to a Sciex (Framingham, MA) 6500 QTRAP mass spectrometer. This system is located in the Metabolomics and Proteomics Laboratory within the Texas Children’s Microbiome Center. Quantitative analysis of metabolites for serotonin pathway (e.g. tryptophan), dopamine pathway (e.g. tyrosine) and glutamine cycle (e.g. GABA, glutamate, and glutamine) was performed using a Phenomenex Luna C18-based analytical column (Torrace, CA, USA), a Restek Raptor C18-based analytical column (Bellafonte, PA, USA) and a Thermo Fisher Accucore fluorophenyl-based analytical column (Waltham, MA, USA), respectively. The MS system was operated in positive ion mode with the TurboIonSpray™ emitter installed in the TurboV ion source. All source voltages, gas settings, mobile phase system, and molecule specific parameters are outlined in S1 Text in S1 File.

### Statistical analysis

Descriptive statistics for independent demographic, clinical, growth and anthropometric, and dietary variables were calculated using MiniTab Statistical Software (v18.0) (Minitab, LLC, State College, PA). Mann-Whitney U tests (also called the Wilcoxon rank sum tests) were performed to detect differences in age, pubertal status, age at menarche, growth and anthropometric measurements, salivary cortisol levels, and dietary nutrient intakes between RTT and control groups. Chi-squared tests were used to detect differences in clinical symptoms and medication use between RTT and control groups.

Wilcoxon rank sum test was used to detect differences in alpha diversity (observed OTUs, ACE index, Fisher’s alpha index of diversity and Faith’s phylogenetic diversity) between RTT and control groups. Permutational multivariate analysis of variance (PERMANOVA), implemented as ‘adonis’ function in the ‘vegan’ R-package (v2.5.6) [43], was used to compare the microbiome composition (beta diversity) between RTT and control groups. A significant PERMANOVA result was evaluated further using a permutation-based homogeneity of multivariate dispersions (PERMDISP) to distinguish whether the difference existed in the average community composition or the variability in the community composition or both.

Pearson’s correlation analysis was performed to test the association of alpha diversity with anthropometric measures and age of RTT participants. PERMANOVA was used on the Bray-Curtis dissimilarity index and UniFrac distance matrices to test the effect of the demographic or clinical factors on the microbiome composition between groups in RTT participants. Additionally, pairwise comparisons of the microbiome composition between CSS groups in RTT cohort was performed using the ‘pairwiseAdonis’ R-package [44].

Differential abundance analysis was performed using the Wilcoxon rank sum test (two groups) or Kruskal-Wallis (more than two groups) coupled with post hoc Dunn test.

The Wilcoxon rank sum test was used to compare fecal metabolite concentrations between RTT and control groups, as well as within the RTT cohort by clinical phenotype (e.g. abdominal distention, puberty).

Statistical analyses were performed primarily in R-software (v4.0.0) [45]. When multiple hypotheses testing were involved, the Benjamini-Hochberg (BH) correction was applied to control the false-discovery rate (FDR). P-values <0.05 were considered significant for all analyses.

## Results

### Characteristics of study participants

The study cohort was comprised of 65 females, 44 girls and young women with RTT, and 21 gender-matched unaffected controls, age range 5–36 y, with race and ethnicity distribution predominately Caucasian (Table 1). The post-pubertal:pre-pubertal ratio was significantly greater in the RTT cohort than in controls, but the ratio of post-pubertal individuals who achieved menarche was similar between both groups. Height, weight, and BMI z-scores were significantly lower in the RTT cohort compared with controls. Triceps skinfold thickness was significantly lower in individuals with RTT compared with controls, but arm muscle area and body fat, expressed as a proportion of body weight, did not differ between groups.

Nearly all individuals in the RTT cohort had mutations in the *MECP2* gene, with the distribution of mutation severity ranging from mild, moderate, to severe (1.4:1.9:1). The distribution of clinical severity scores (CSS), ranging from mild, moderate, to severe, was 1.2:1.8:1. Reported symptoms of anxiety, bruxism, hyperventilation, bloating, and seizures were significantly more frequent in the RTT cohort than in controls. Additionally, RTT individuals were significantly more anxious, measured by salivary cortisol levels, than controls. The use of medications including proton pump inhibitors, prokinetics, probiotics, laxatives, and anticonvulsants, but not birth control pills was significantly more frequent in the RTT cohort than in controls. The GHQ scores across modules related to gastrointestinal health and function, mood and behavior, and parental concerns were significantly greater among RTT individuals compared with controls. More than one-third of the RTT cohort was non-ambulatory.

Approximately one-half of the RTT cohort consumed table food and commercial formulas combined, whereas approximately one-fourth each consumed table food or formula alone as the sole source of their dietary intake (Table 2). Three-fourths of the RTT cohort received multivitamin and/or mineral supplements. Although dietary carbohydrate and fat intakes, expressed as proportions of dietary energy intake, did not differ between RTT and control groups, total daily dietary energy and fiber intakes were significantly lower in RTT individuals than in controls. Dietary protein and B complex vitamin intakes were similar between groups with the exception of riboflavin and cobalamin. Dietary intakes of selected amino acids including tryptophan, phenylalanine, tyrosine, leucine, methionine were similar between the RTT cohort and controls, but dietary glutamate intakes were significantly lower in the RTT group. Plasma concentrations of phenylalanine, tyrosine, leucine, methionine, glutamate, and glutamine were within the normal range, but plasma glutamate (33 vs 44 umol/L, p<0.06) and glutamine (531 vs 618 umol/L, p<0.09)) concentrations tended to be lower in RTT individuals affected with abdominal distention (n = 22) compared with RTT individuals without abdominal distention (n = 22), respectively. Plasma phenylalanine (45 vs 45 μmol/L, NS) and tyrosine (56 vs 65 μmol/L, NS) concentrations did not differ between respective groups.

**Table 2:** Dietary intakes of participants with RTT and unaffected controls.

Features	Group of Individuals*		p-value
	RTT	C	
*Dietary Source*			
Number of subjects	44	21	-
Food (%)	73	100	0.001
Formula (%)	75	0	0.001
Multivitamins and/or mineral supplements (%)	75	24	0.001
*Dietary Intake*			
Number of participants	41	19	-
Macronutrients			
Energy (kcal/d)	1313 (433, 2402)	1659 (878, 2035)	0.05
Fat (% E)	34 (18, 85)	35 (26,50)	NS
Carbohydrate (% E)	52 (4, 70)	50 (25, 62)	NS
Protein (g/d)	44 (21, 108)	56 (23, 91)	NS
B-Complex Vitamins			
Thiamine (mg/d)	0.9 (0, 3.8)	1.2 (0.5, 2.7)	NS
Riboflavin (mg/d)	2.2 (0.6, 9.7)	1.3 (0.7, 3.9)	0.01
Niacin (mg/d)	28 (13, 94)	27 (14, 45)	NS
Folate (mcg/d)	333 (0, 963)	314 (92,630)	NS
Cobalamin (mcg/d)	6.1 (1.3, 15.9)	3.0 (1.3, 10.2)	0.001
Fiber (g/day)	6.2 (3.4, 26.9)	11.1 (3.4, 26.9)	0.01
Amino Acids			
Tryptophan (g/day)	0.60 (0, 1.7)	0.61 (0.3, 1.1)	NS
Leucine (g/day)	4.11 (0.1, 9.1)	4.30 (2.2, 6.5)	NS
Methionine (g/day)	1.12 (0, 2.4)	1.31 (0.8, 2.0)	NS
Phenylalanine (g/day)	1.87 (0.1, 4.5)	2.33 (1.2, 3.9)	NS
Tyrosine (g/day)	1.72 (0, 3.7)	1.92 (1.0, 3.0)	NS
Glutamate (g/day)	8.16 (0, 19.4)	9.89 (5.8, 15.2)	0.04
Plasma amino acids			
Leucine (μmol/L)	81 (40, 222)	-	-
Methionine (μmol/L)	20 (8, 69)	-	-
Phenylalanine (μmol/L)	45 (25, 87)	-	-
Tyrosine (μmol/L)	56 (27, 167)	-	-
Glutamate (μmol/L)	36, (12, 89)	-	-
Glutamine (μmol/L)	567 (295, 856)	-	-

*RTT, Rett; C, control group. Values expressed as median (minimum, maximum) or proportion (%). Mann-Whitney test (continuous variables) or Chi-squared test (discrete variables), p-value <0.05 considered significant.

### Gut microbiome in RTT individuals and unaffected controls

A total of 65 stool samples were sequenced for the V4 region of the bacterial 16S rRNA gene. Three samples (two RTT, one unaffected) were removed from the data set due to fewer than 1000 reads post quality filtering, leaving 62 samples for downstream analyses. Both negative controls, sequenced to monitor the potential background contamination, also were excluded from further analysis because one of them had zero reads after quality filtering while another had fewer than 100 reads post quality steps. An average of 34,463 ± 16,487 reads was retained for each stool sample after quality filtering (S1 Table in S1 File). The rarefaction curves of bacterial richness (observed OTUs) plotted as a function of the sampling effort (sample sequencing depth) suggests that the majority of the samples had sufficient reads to capture most of the community diversity (S1 Fig in S1 File).

**Fig 1:**
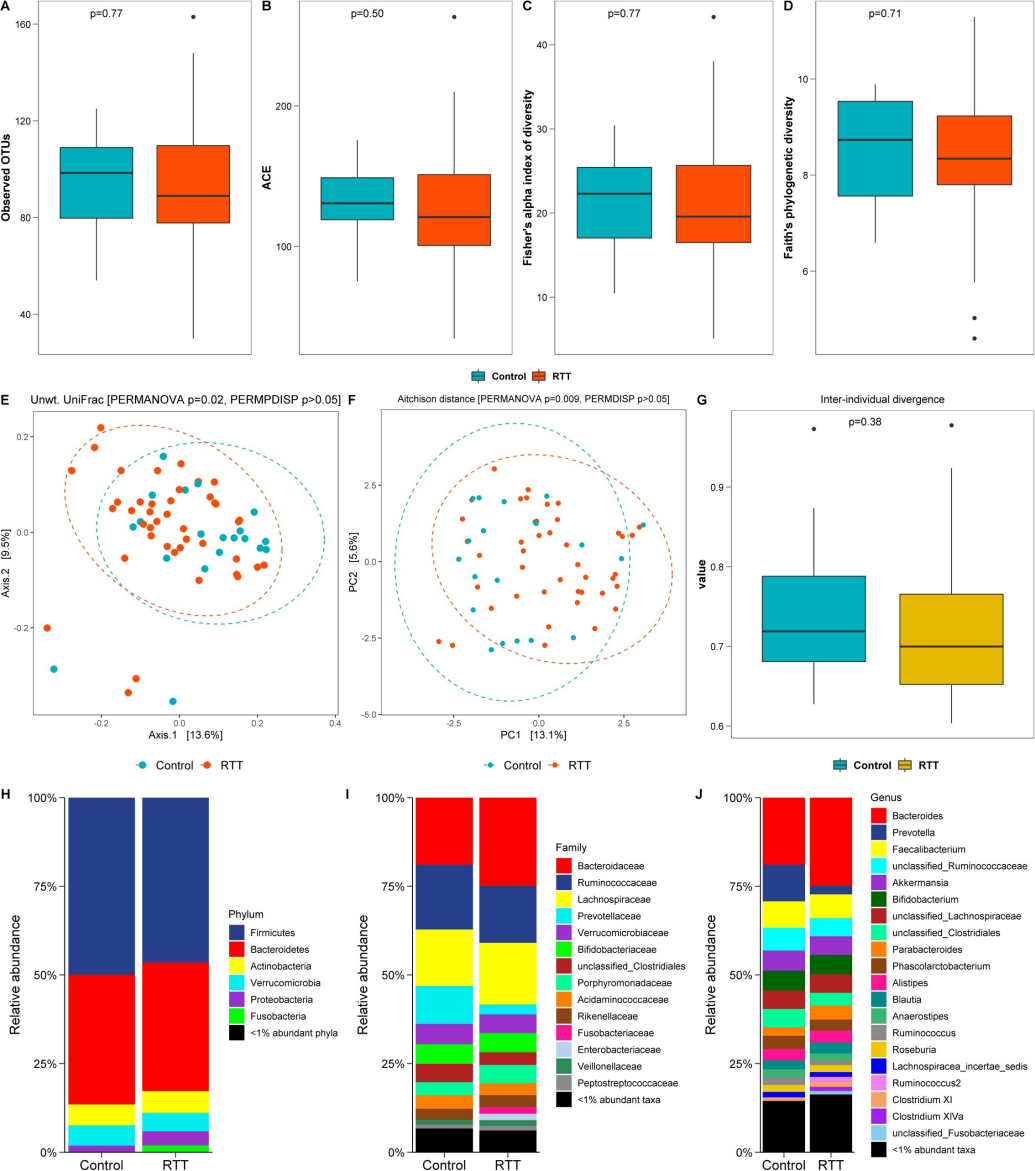
Gut bacterial microbiome in RTT patients and unaffected controls. Number of observed OTUs (**A**), abundance based coverage estimate (ACE) values (**B**), Fisher’s alpha index of diversity (**C**) and Faith’s phylogenetic diversity (**D**) were slightly lower, but not statistically significant, in the RTT group than the control. Wilcoxon rank sum p <0.05 was considered statistically significant. Beta diversity ordinations using PCoA plot of the unweighted UniFrac distance (**E**) and PCA plot of the Aitchison distance (**F**) showed differences in bacterial composition between the RTT and control groups (PERMANOVA p<0.05). Axis labels represent the percentage variation explained by each axis. Unwt. = unweighted; PCo = principal coordinate; PC = principal component. The first two coordinates/components that explained the largest fraction of variably in our data was plotted. The inter-individual divergence value with respect to the median profile (**G**) within the control group was generally larger than the RTT group. Wilcoxon rank sum test p <0.05 was considered statistically significant. **H**-**J** shows relative abundance of bacterial taxa in the RTT and control groups at various taxonomic levels.

Both observed richness (measured by observed OTUs, Fig 1A) and estimated richness (abundance based coverage estimate (ACE) value, Fig 1B) of the bacterial community in the RTT group were not significantly different than the control group. Similarly, Fisher’s alpha (Fig 1C) and Faith’s phylogenetic diversity (Fig 1D) indices were not significantly different between the RTT group and control group.

Beta diversity ordination using the unweighted UniFrac distance metrics (Fig 1E) revealed a significant difference in bacterial composition between RTT and control groups. However, bacterial composition using the Bray-Curtis dissimilarity index and weighted UniFrac distance metrics did not differ between RTT and control groups (S2 Fig in S1 File). The unweighted PCoA plot showed some separation between RTT and control samples, with a fair degree of overlap, but no difference in dispersion (or spread) of samples within each group (Fig 1E). Similar to the unweighted UniFrac result, principal component analysis (PCA) of the Aitchison distance using centered log-ratio (CLR) transformed data also found a significant difference in the community composition between RTT and control groups, with no effect of sample dispersion (Fig 1F). The control group in general had a relatively more heterogeneous community composition (i.e. higher divergence with respect to the median profile) than the RTT group (Fig 1G).

The mean relative abundance (%) of bacterial taxa at the phylum (Fig 1H), family (Fig 1I), and genus (Fig 1J) levels was not significantly different between RTT and control groups. The predominant phyla [mean relative abundance (%)] in RTT and control (C) groups were Firmicutes (RTT: 46.5, C: 49.9) and Bacteroidetes (RTT: 36.3, C: 36.6). The predominant families [mean relative abundance (%)] were Bacteroidaceae (RTT: 24.9, C: 18.9), Lachnospiraceae (RTT: 17.4, C: 15.9), Ruminococcaceae (RTT: 16.1, C: 18.3), Bifidobacteriaceae (RTT: 5.4, C: 5.6), Verrucomicrobiaceae (RTT: 5.2, C: 5.7), Porphyromonadaceae (RTT: 5.1, C: 3.7) and Prevotellaceae (RTT: 2.8, C: 10.7). Although the genera [mean relative abundance (%)] *Bacteroides* (RTT: 24.9, C: 18.9), *Parabacteroides* (RTT: 4, C: 2.4), and *Clostridium* XlVa (RTT: 1.2, C: 0.9) were higher, and *Prevotella* (RTT: 2.4, C: 10.3) and *Faecalibacterium* (RTT: 6.7, C: 7.5) were lower in the RTT group compared with the control group, these values did not achieve significance after FDR correction. Because we did not find differences in the gut microbiome between individuals with RTT and controls, we focused further analyses only in the RTT group.

### Effects of clinical phenotype on the gut microbial community in RTT

The age of the RTT cohort tended to correlate inversely with alpha diversity of the gut microbiome, but this association did not achieve significance (S3 Fig in S1 File). Height and weight, but not BMI, of the RTT individuals correlated inversely (p<0.05) with alpha diversity (S4 Fig in S1 File). Age, race, BMI, mutation severity, clinical symptoms (anxiety, seizure, bruxism, hyperventilation, abdominal distension), bowel movement frequency, stool consistency, and medication use had no effect on the microbiome composition (beta diversity) in the RTT cohort. However, the microbial community composition in the RTT cohort was significantly different based on pubertal status, a surrogate for age, and the clinical severity score (CSS) (Table 3).

**Table 3:** Effects of various factors on the gut microbiome composition in RTT, as measured by PERMANOVA test of Bray-Curtis dissimilarity index and UniFrac distance matrices.

Variable	Group	Bray-Curtis			Unweighted UniFrac			Weighted UniFrac		
		F	R^2^	P	F	R^2^	P	F	R^2^	P
***Demographic and Anthropogenic factors***										
**Age group**	Pediatric (n = 17), adolescent (n = 13), adult (n = 12)	1.11	0.05	0.262	1.06	0.05	0.331	1.29	0.06	0.186
**Pubertal status**	Pre-puberty (n = 9), post-puberty (n = 33)	1.70	0.04	0.018*	1.37	0.03	0.078	2.86	0.07	0.003*
**Menses**	Yes (n = 21), no (n = 21)	0.69	0.02	0.926	1.16	0.03	0.198	0.86	0.02	0.550
**Birth control pills**	Yes (n = 7), no (n = 34)	1.11	0.03	0.298	1.18	0.03	0.188	1.18	0.03	0.276
**Race**	White (n = 23), Black (n = 5), Hispanic (n = 12)	0.98	0.05	0.468	0.99	0.05	0.432	0.82	0.04	0.680
**BMI z-score**	Normal ≥ -1 (n = 28), abnormal < -1 (n = 14)	0.99	0.02	0.445	1.14	0.03	0.235	0.98	0.02	0.429
***Mutation and clinical phenotypes***										
**Mutation severity**	Mild (n = 12), moderate (n = 10), severe (n = 10)	0.93	0.05	0.652	0.78	0.04	0.937	1.04	0.05	0.412
**Anxiety**	Yes (n = 22), no (n = 20)	0.96	0.02	0.511	0.97	0.02	0.478	0.44	0.01	0.953
**Bruxism**	Yes (n = 28), no (n = 14)	0.75	0.02	0.861	0.96	0.02	0.495	0.89	0.02	0.526
**Seizure**	Yes (n = 27), no (n = 15)	0.90	0.02	0.629	1.13	0.03	0.241	0.84	0.02	0.589
**Hyper-ventilation**	Yes (n = 28), no (n = 14)	0.89	0.02	0.641	0.99	0.02	0.478	0.90	0.02	0.514
**Abdominal distention**	Distention (n = 21), no distention (n = 21)	0.81	0.02	0.801	0.99	0.02	0.473	0.87	0.02	0.551
**Stool frequency**	<1 stool/day (n = 16), ≥1 stool/day (n = 26)	0.79	0.02	0.802	1.00	0.02	0.424	0.81	0.02	0.599
**Stool consistency**	Loose (diarrhea) (n = 25), normal (n = 10), formed (constipated) (n = 7)	1.21	0.06	0.117	1.07	0.05	0.294	1.25	0.06	0.186
**Clinical severity score**	Mild (n = 10), moderate (n = 18), severe (n = 10)	1.30	0.07	0.068	1.41	0.07	0.019*	1.03	0.06	0.411
***Medications***										
**Proton pump inhibitors**	Yes (n = 27), no (n = 15)	0.94	0.02	0.530	0.80	0.02	0.823	1.07	0.03	0.365
**Prokinetics**	Yes (n = 8), no (n = 34)	0.87	0.02	0.671	0.76	0.02	0.877	0.71	0.02	0.702
**Probiotics**	Yes (n = 10), no (n = 32)	0.94	0.02	0.539	0.85	0.02	0.717	1.00	0.02	0.407
**Laxatives**	Yes (n = 30), no (n = 12)	0.93	0.02	0.583	0.77	0.02	0.862	0.91	0.02	0.482
**Anticonvulsants**	Yes (n = 25), no (n = 17)	1.08	0.03	0.336	1.02	0.02	0.425	0.79	0.02	0.667
**Vagal nerve stimulation therapy**	Yes (n = 6), no (n = 36)	0.95	0.02	0.532	0.77	0.02	0.826	1.24	0.03	0.255
***Diet***										
**Food and formula**	Food only (n = 11), formula only (n = 12), food + formula (n = 19)	1.60	0.08	0.006*	2.04	0.09	0.001*	2.53	0.11	0.002*
**Food vs formula**	Food only (n = 11), formula only (n = 12)	1.85	0.08	0.009*	2.09	0.09	0.002*	1.76	0.08	0.043*
**Energy^$^**	Low (n = 15), high (n = 24)	1.26	0.03	0.146	1.41	0.04	0.065	1.55	0.04	0.120
**Fiber^$ $^**	Low (n = 15), high (n = 24)	1.82	0.05	0.006*	1.68	0.04	0.011*	1.12	0.03	0.31
**Glutamate^$ $ $^**	Low (n = 19), high (n = 20)	1.11	0.03	0.285	1.29	0.03	0.126	0.75	0.02	0.668
**Vitamin B12^€^**	Low (n = 22), high (n = 17)	0.90	0.02	0.592	0.75	0.02	0.881	0.62	0.02	0.822

F = F-value, R^2^ = effect size, p = p-value

*statistically significant; BMI = body mass index

^$^daily energy intake of <1146 kcal was considered low

^$ $^fiber intake of <4.1 g/day was categorized as low

^$ $ $^intake of <7.6 g Glutamate/day was considered low

^**€**^vitamin B12 (Cobalamin) intake of >6.2 mg/day was categorized as high.

### Pubertal status and the microbiome in RTT

**Fig 2:**
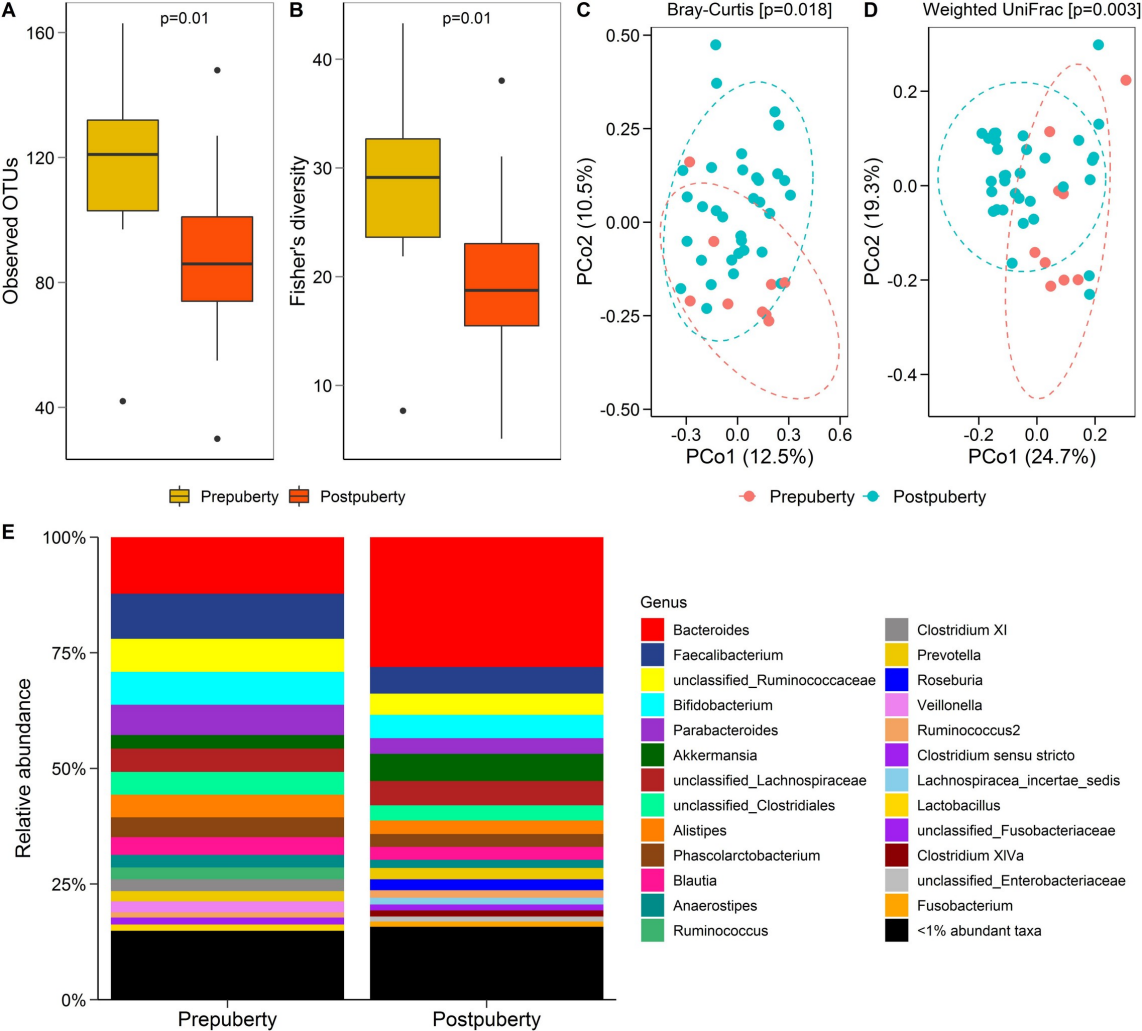
Gut bacterial diversity and composition in subjects with Rett syndrome (RTT) by pubertal status. Observed OTUs (**A**) and Fisher’s alpha index of diversity (**B**) in the RTT cohort by pubertal status (Wilcoxon ranks sum test). PCoA ordinations of Bray-Curtis dissimilarity (**C**) and weighted UniFrac distance (**D**) measures in pre-puberty and post-puberty groups in RTT cohort. Genus level taxonomic summary of bacteria in pre- and post-pubertal groups in RTT patients (**E**).

Bacterial richness (observed OTUs) (Fig 2A) and diversity (Fisher’s alpha index) (Fig 2B) decreased significantly from pre- to post-puberty in the RTT cohort. PCoA ordinations of Bray-Curtis dissimilarity (Fig 2C) and weighted UniFrac distance (Fig 2D) measures also revealed a significant difference in the community composition between pre- and post-puberty in the RTT cohort. However, the relative abundance (%) of the bacterial taxa (Fig 2E) was not significantly different among RTT individuals based on pubertal status, with the exception of one taxon, “unclassified Desulfovibrionales”.

### Clinical severity score and the microbiome in RTT

**Fig 3:**
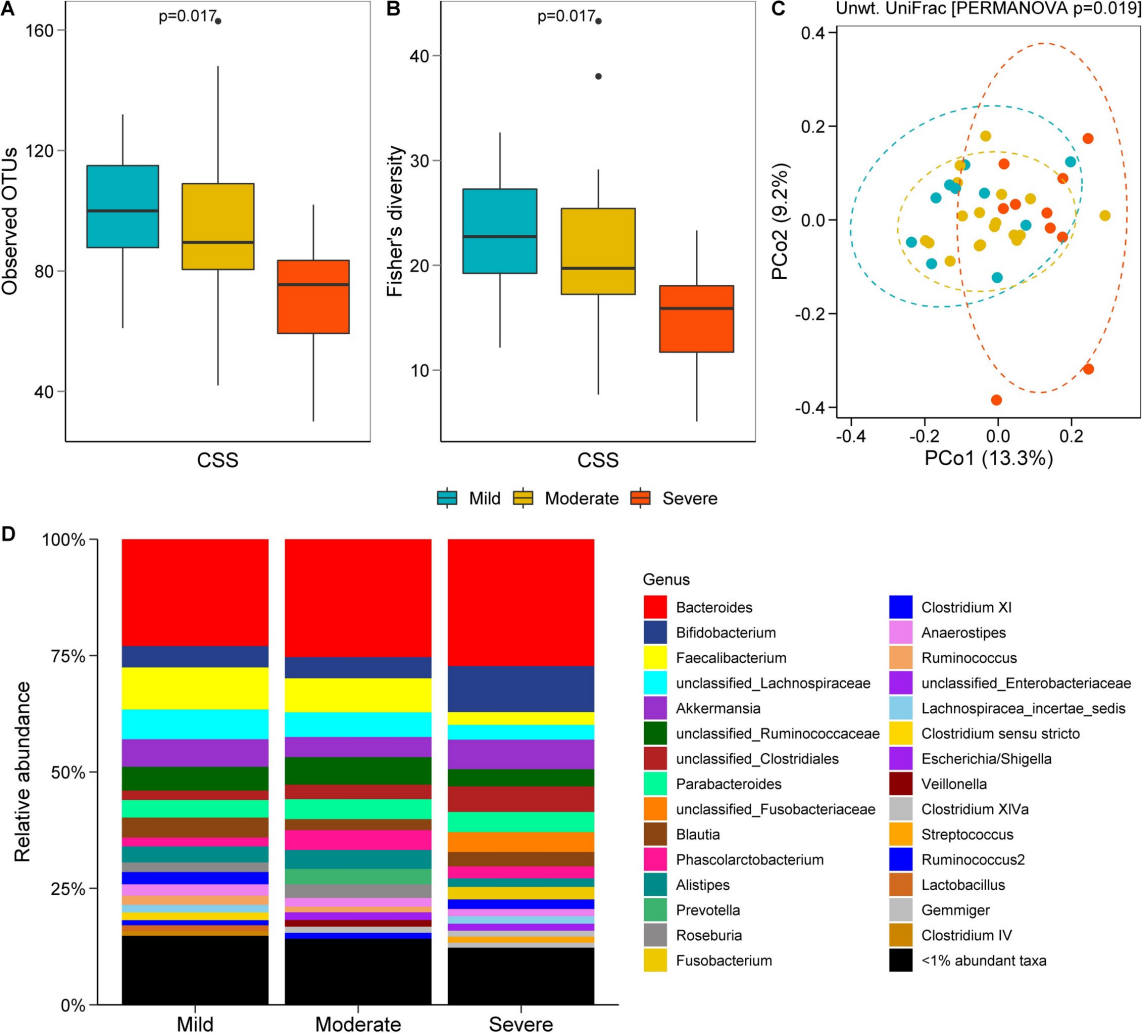
Gut bacterial richness, diversity, and composition in individuals with Rett syndrome (RTT) by Clinical Severity Score (CSS). Number of observed OTUs (**A**) and Fisher’s alpha index of diversity (**B**) by CSS. PCoA plot of unweighted UniFrac distance (**C**) based on CSS groups. Relative abundance (%) of bacterial taxa at the genus level (**D**) in the RTT cohort grouped by CSS. Mild (CSS ≤19, n = 10), moderate (CSS = 20–30, n = 18) and severe (CSS ≥31, n = 10).

RTT individuals with a severe (CSS ≥31) score had a less rich (Fig 3A) and less diverse (Fig 3B) gut microbiome compared to those who had a mild (CSS ≤19) or moderate (CSS 20–30) score. Unweighted UniFrac-based beta diversity analysis revealed that the gut bacterial community of RTT individuals with severe disease was distinct from those with a mild or moderate CSS (Fig 3C). The effect of CSS on the beta diversity was weak (R^2^ = 0.07), but significant, when all three CSS groups were compared (Fig 3C), possibly because of a wider dispersion of samples in the moderate CSS group (S5 Fig in S1 File). Pairwise PERMANOVA tests further revealed that the microbiome composition in mild and moderate CSS groups was significantly different than the severe CSS group, but not the composition between mild and moderate groups (S2 Table in S1 File). The relative abundance (%) of bacteria did not differ in RTT individuals when the CSS groups (mild, moderate, severe) were compared separately (Fig 3D).

### Diet and the microbiome in RTT

Bacterial richness, measured by observed OTUs (S6A and S6C Fig in S1 File), was higher in RTT individuals who consumed table foods alone or combined with formula compared with those who received formula as their sole dietary source. Similar results were obtained with bacterial diversity as determined by the Fisher’s alpha index (S6B and S6D Fig in S1 File). Bacterial composition (beta diversity), measured by both Bray-Curtis dissimilarity and unweighted UniFrac distance metric, differed significantly between RTT individuals who consumed table food and formula (Table 3). However, the relative abundance (%) of bacteria was not significantly different among the groups of RTT individuals when categorized by their dietary food and formula consumption (S6E Fig in S1 File) with the exception of a rare taxon, “unclassified_Firmicutes”. Overall, genus *Bifidobacterium* was increased in formula consuming patients. The formulas administered to the participants included: a whole cow milk protein source (58% of affected participants), a protein hydrolysate source (19% of affected participants), a soy protein source (3% of affected participants), an amino acid source (8% of affected participants), and an alternative plant, nut, and/or meat based source (11% of affected participants).

Bacterial composition (beta diversity) was significantly different based on whether RTT individuals consumed more than or less than 4.1 g fiber daily (Table 3 and S7 Fig in S1 File), but not on daily dietary intakes of energy (1146 kcal or less), glutamate (7.6 g or less), or vitamin B12 (6.2 mg or less).

### Metabolomic profiles in RTT individuals and unaffected controls

**Fig 4:**
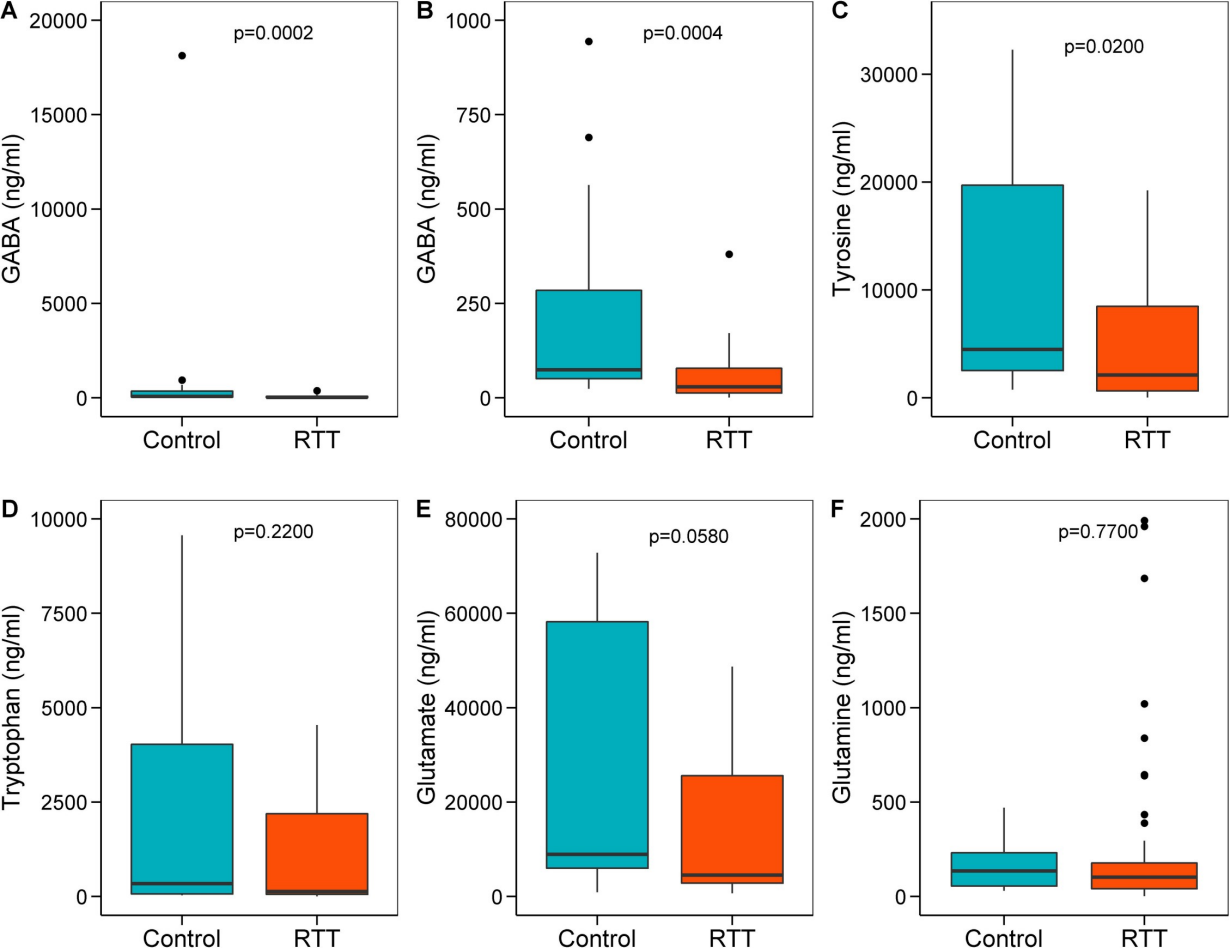
Comparisons of gut metabolites in individuals with Rett syndrome (RTT) and unaffected Controls (C). Box and whisker plots showing the fecal concentrations (ng/ml) of GABA (**A, B**), tyrosine (**C**), tryptophan (**D**), glutamate (**E)** and glutamine (**F**) in RTT and control groups. Fig **B** shows the difference in fecal GABA content between RTT and C after removing an outlier in the C group. Data refer to fecal supernatant as measured using LC-MS. RTT, n = 42; C, n = 20. Wilcoxon rank sum test p<0.05 was considered significant.

Fecal GABA (Fig 4A and 4B) and tyrosine (Fig 4C) concentrations were significantly lower and fecal glutamate (Fig 4E) tended to be lower in the RTT cohort compared with controls. Fecal concentrations of tryptophan (Fig 4D) and glutamine (Fig 4F) did not differ between RTT and control groups.

## Discussion and conclusions

Gastrointestinal problems affect the health and quality of life in individuals with RTT and may be more debilitating than the underlying neurological features of the disorder. Dysbiosis of the gut microbiome may contribute to the gastrointestinal manifestations in affected individuals. In the present study, we demonstrated that the gut bacterial microbiome differed significantly within the RTT cohort based on the pubertal status and CSS of the individuals and the type of diet they received. Although the composition of the gut microbiome did not differ between affected and unaffected individuals, concentrations of selected protein end-products of the gut bacterial metabolome, including GABA, tyrosine, and glutamate, were lower in the RTT cohort. At present, we cannot infer causality between gut bacterial dysbiosis and gastrointestinal dysfunction, but alterations in the gut metabolome may provide clues to the pathophysiology of gastrointestinal problems in RTT.

Functional gastrointestinal disorders that manifest symptoms such as bloating, altered bowel habits, and abdominal pain commonly affect children of all ages, including those with RTT [4, 46]. Increasing evidence supports involvement of gut microbes in functional bowel disorders [47]. Healthy children with irritable bowel symptoms, predominantly abdominal pain, demonstrate enrichments of several bacterial taxa including the genera *Flavonifractor*, Lachnospiraceae, *Alistipes*, *Akkermansia*, and *Parabacteroides* [47, 48]. In contrast, few studies have characterized the gut microbiome in relation to gastrointestinal symptoms in RTT [23, 24]. In one Italian RTT cohort, altered microbial composition, characterized by α- and β-diversity and relative abundance at the genus level of taxonomy, was identified between individuals with RTT and unaffected individuals, based on the presence or absence of constipation, respectively, but not between RTT individuals with or without constipation [23]. Contrary to the Italian report [23], we did not find differences in α- or β-diversity, nor the relative abundance of any taxa, between RTT and unaffected individuals. Moreover, we were unable to detect differences in the microbiome composition within the RTT group, based on the presence or absence of symptoms, including anxiety, hyperventilation, abdominal distention, or changes in stool frequency and consistency. The explanation for these differences between studies is not readily apparent, although it is well-recognized that the gut microbiome, even among healthy individuals, may vary due to differences in age [25], ethnicity [49], geography [50], dietary habits [51], life-style differences [27, 28] or environment [52]. These findings suggest that increased sampling from a broad, diverse population will be necessary to appreciate fully the dysbiosis of the gut microbiome between RTT and unaffected individuals [53].

Dysbiosis in gastrointestinal disorders is characterized by an expansion or reduction of members across taxa, along with altered community richness [53, 54]. In children with the irritable bowel syndrome, enrichments in the abundance of *Flavonifractor*, Lachnospiraceae, *Dorea*, *Ruminococcus*, and *Clostridium* spp. and reductions in the abundance of *Bifidobacterium* and *Faecalibacterium* have been described [48, 55]. In children with inflammatory bowel disease, a reduction in the abundance of *Bacteroides*, *Faecalibacterium*, *Roseburia*, *Blautia*, *Ruminococcus*, and *Coprococcus* has been associated with Crohn disease [53]. More specifically, a reduction in the ileal abundance of *Faecalibacterium prauznitzii*, an anti-inflammatory bacterium considered a marker of bowel health, correlates with a higher rate of disease recurrence in inflammatory bowel disease [56]. In RTT, the pattern of the microbiome varies. In the large Italian RTT cohort, *Bifidobacterium* was the predominant genus [23], whereas in the small Italian RTT cohort, the gut microbiome was enriched by two genera, *Bacteroides* and *Clostridium* [24]. In the present RTT cohort, the overall pattern of the microbiome, in particular, a higher relative abundance of *Bacteroides* spp. and *Clostridium* spp. and lower relative abundance of *Prevotella* spp. and *Faecalibacterium* spp., suggest a change in the gut bacterial composition towards an inflammatory, dysbiotic community [56, 57].This dysbiotic pattern also has been reported in the autism spectrum disorder, another neurodevelopmental disorder [30]. An increased abundance of *Bacteroides* is particularly bothersome because this genus contains a number of clinical pathogens found in anaerobic infections associated with a high mortality rate [58]. Furthermore, a reduction in the abundance of *Faecalibacterium*, a genus known to produce short-chain fatty acids (e.g., butyrate) that strengthen gut barrier function and protect against inflammation [59], supports a pro-inflammatory status of the gut microbiome in RTT [60].

In the present study, we found two biomarkers of dysbiosis, pubertal status and clinical severity of RTT. Increasing biologic age, with puberty as a specific inflexion point, is a known biomarker of increased gut microbial diversity [50]. However, a reduction in bacterial richness and diversity in the post-puberty RTT group compared with that of the pre-pubertal group suggests an inverse response of the intestinal microbiome based on pubertal timing in adolescence. Although we did not identify changes in specific taxa, a report in a cohort of girls at risk for allergy found that the time of peak height velocity, a marker of pubertal maturation, correlated positively with the relative abundance of the taxon, *Gemella*, and negatively with the relative abundance of the taxon, *Barnesiella* [61]. In contrast, a study in healthy Chinese females demonstrated that the taxon, *Lactococcus*, was more prevalent during pre-puberty whereas the taxa *Parabacteroides*, *Phascolarctobacterium*, *Alistipes*, and *Oscillospira* were dominant post-puberty [62]. In addition, we found a reduction in gut microbial diversity with increased severity of RTT, a pattern common to other gastrointestinal disorders such as inflammatory bowel disease [63]. The Italian study of the small RTT cohort also reported lower bacterial diversity in individuals with more severe symptoms [24]. This latter finding begs further investigation of key modulator microbes that affect the severity of RTT, suggesting that it may be possible to predict the severity of symptom progression based on the gut microbiome composition.

Diet has a significant impact on the composition and function of the gut microbiome [64, 65]. A *Bacteroides*-enriched enterotype is characteristic of a high-protein, high-saturated fat Western diet, whereas a *Prevotella*-enriched enterotype is characteristic of a high carbohydrate, fiber-rich Mediterranean diet [66]. Dietary modifications such as exclusive enteral formula feedings and diets with a reduction in fermentable oligo-, di-, monosaccharides, and polyols (FODMAP) have been employed to reshape the gut microbiome for the relief of symptoms associated with inflammatory bowel disease and the irritable bowel syndrome, respectively [67–69]. Our findings of higher bacterial richness and α-diversity among RTT individuals who consumed table foods compared with those who primarily received formula suggest a potential benefit of table foods, particularly a vegetable-rich, fiber-rich diet [70]. Differences in beta diversity (bacterial composition) among RTT individuals who consumed dietary fiber in amounts less than or more than 4.1 g/day suggest possible microbial signatures for this dietary component. Diets rich in fiber have been associated with an increased abundance of beneficial microbes including *Bifidobacterium* spp. and *Lactobacillus* spp., as well as higher fecal butyrate concentrations [71] and the relative abundance of the flagellin protein [70]. In the RTT cohort, *Bacteroides* was the predominant genus among all diet groups, consistent with findings of increased protein consumption typical of Western diets [66]. However, the genus *Prevotella*, found in association with high-carbohydrate, high-fiber diets, was prominent in RTT individuals who consumed table food, whereas the genus *Bifidobacterium*, was prominent in RTT individuals who received formula as their primary food source. Finally, the differences in the estimates of dietary glutamate and glutamine consumption and the fecal concentrations of GABA and glutamate between the RTT cohort and unaffected individuals were of interest to us. The lower levels of dietary glutamate consumption and fecal glutamate concentrations, particularly in the context of the positive association between low plasma glutamate and glutamine concentrations and the presence of abdominal distention in RTT individuals, offers a potential intervention strategy for gastrointestinal symptoms. Altered levels of glutamate were identified similarly in the serum metabolomic analysis of individuals with fibromyalgia, suggesting changes in neurotransmitter metabolism [72]. In addition, germ-free mice receiving fecal transplants from individuals with schizophrenia demonstrated altered glutamate, glutamine, and GABA concentrations in the hippocampus, suggesting aberrant neurochemistry and neurologic function [73].

The limitations of this study are primarily those of small sample size and site of microbe sampling. The creation of a large multicenter cohort of children with inflammatory bowel disease demonstrated enhanced resolution and statistical power for studying the role of the microbiome in this disorder when sample size ranged from 200 to 400 individuals per group [53]. Although our sample size was reasonably robust, in the context of confounding factors such as age, clinical severity of RTT, and diet, a much larger sample size will be necessary to detect differences in relative abundance of bacterial taxa between affected and unaffected individuals. Capturing microbial shifts in their full complexity requires much larger study designs to optimize outcomes. In addition, it is clear that the gut microbiome differs based on the sampling site. In children with inflammatory bowel disease, specifically Crohn disease, dysbiosis in the gut microbial community was observed only in the microbiome profiles obtained from tissue samples, not from stool samples [53]. This observation implies that a dysbiotic state is less evident in the gut lumen despite symptoms, explaining the potential lack of a biomarker signature and emphasizing the need to examine tissue samples to gain a better understanding of the pathophysiology of gastrointestinal dysmotility in RTT.

In conclusion, our findings provide benchmark information about the gut bacterial community in RTT and confounding factors that influence its composition. Further studies using a multi-omics approach may provide new information about the functional aspects of the RTT phenotype, diet, and gut microbiome interactions in individuals with RTT and may reveal targets for manipulation of the gut microbiome for prevention or management of the gastrointestinal complications of this disorder.

## Supporting information

S1 File (PDF)
