# Supplementary material for: Assessment of the gut bacterial microbiome and metabolome of girls and women with Rett Syndrome
Source: PLoS One. 2021 May 6;16(5):e0251231. doi: 10.1371/journal.pone.0251231 (PMC8101921; doi:10.1371/journal.pone.0251231)
Supplement: S1 File — (PDF) [file pone.0251231.s001.pdf]

S1 File

Assessment of the gut bacterial microbiome and metabolome of girls and women with Rett Syndrome

Santosh Thapa[1,2], Alamelu Venkatachalam[1,2], Nabeel Khan[3], Mohammed Naqvi[3,#a], Miriam Balderas[1,2], Jessica K. Runge[1,2], Anthony Haag[4], Kathleen M. Hoch[4], Daniel G. Glaze[3,5], Ruth Ann Luna[1,2], Kathleen J. Motil[3,6*]

[1]Medical Metagenomics Laboratory, Texas Children's Microbiome Center, Department of Pathology, Texas Children's Hospital, Houston, Texas, United States of America

[2]Department of Pathology and Immunology, Baylor College of Medicine, Houston, Texas, United States of America

[3]Department of Pediatrics, Baylor College of Medicine, Houston, Texas, United States of America

[4]Metabolomics and Proteomics Laboratory, Texas Children's Microbiome Center, Department of Pathology, Texas Children's Hospital, Houston, Texas, United States of America

[5]Department of Neurology, Baylor College of Medicine, Houston, Texas, United States of America

[6]USDA/ARS Children's Nutrition Research Center, Baylor College of Medicine, Houston, Texas, United States of America

[#a]Current Address: Department of Investigational Cancer Therapeutics, The University of Texas MD Anderson Cancer Center, Houston, Texas, United States of America

*Correspondence: kmotil@bcm.edu (KJM)

List of supporting information

S1 Table. 16S v4 RNA read summary post quality filtering.

S1 Fig. Alpha rarefaction curves for bacterial richness plotted against the sequencing depth of stool samples from individuals with Rett syndrome (RTT) and unaffected controls (C).

S2 Fig. Principal coordinate analysis (PCoA) plots of Bray-Curtis dissimilarity and weighted UniFrac distance for bacterial communities in individuals with Rett syndrome (RTT) and unaffected controls (C).

S3 Fig. Correlation between age and alpha diversity of the gut microbiome in individuals with Rett syndrome (RTT).

S4 Fig. Correlation between anthropometric measures and alpha diversity of the gut microbiome in individuals with Rett syndrome (RTT).

S2 Table. Pairwise PERMANOVA tests of unweighted UniFrac distance matrix in RTT patients by CSS groups.

S5 Fig. Dispersion of samples in RTT subjects by CSS groups using unweighted UniFrac distance.

S6 Fig. Gut bacterial diversity and composition in individuals with Rett syndrome (RTT) by dietary source (table food vs formula).

S7 Fig. Gut bacterial composition (beta diversity) in individuals with Rett syndrome (RTT) by dietary fiber source.

S1 Text: Detailed protocol used for the targeted metabolomics.

S1 Table. 16S v4 RNA read summary post quality filtering.

Features	Number (N = 65)	Number (N = 62*)
Minimum number of reads/sample	3	1824
Maximum number of reads/sample	81,323	77,364
Average number of reads/sample ± SD	33,789 ± 18,133	34,463 ± 16,487
Total number of reads	2,196,302	2,136,713
Total number of OTUs	-	962
Number of singletons** OTUs	-	0

*N= number of stool samples after quality filtering and removing unclassified reads at the kingdom and phylum levels, as well as the cyanobacterial and mitochondrial sequences.

**=only one read detected across all samples.

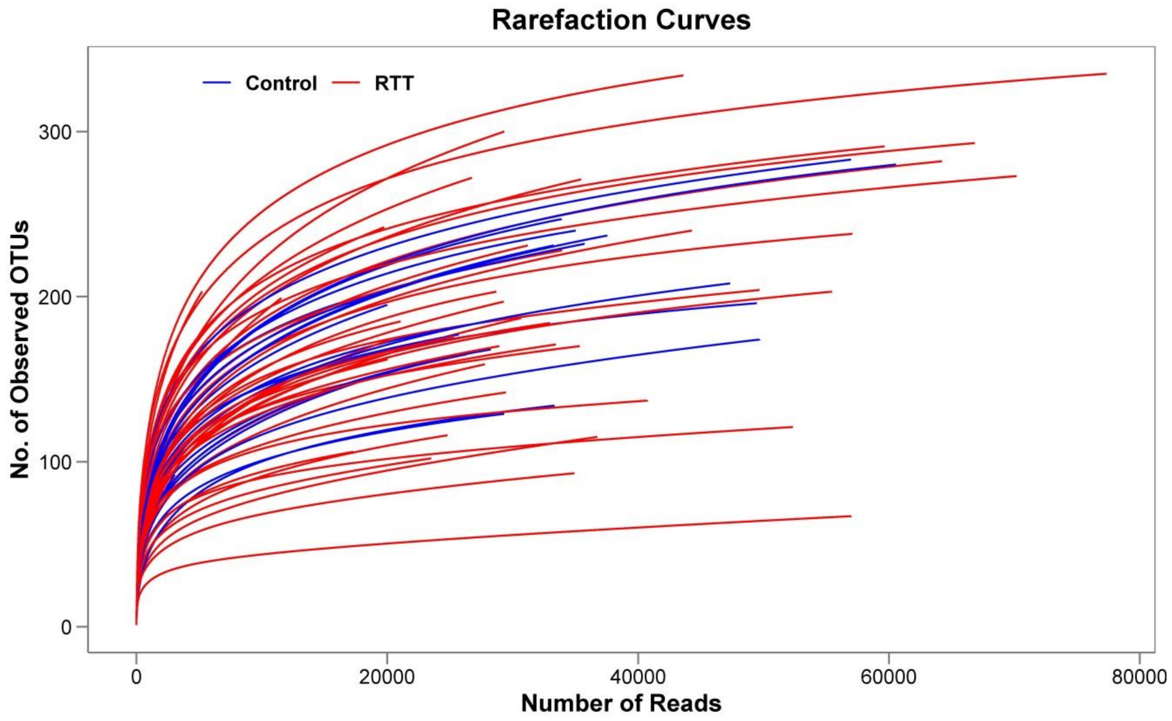

S1 Fig. Alpha rarefaction curves for bacterial richness plotted against the sequencing depth of stool samples from individuals with Rett syndrome (RTT) and unaffected controls (C). Rarefaction curves were generated using *ggrare* function in the *ranacapa* (v0.1.0) R-package to estimate whether all diversity of the microbial community was captured. Each curve represents a sample and shows the number of OTUs found in a random subset of different numbers of sequencing reads.

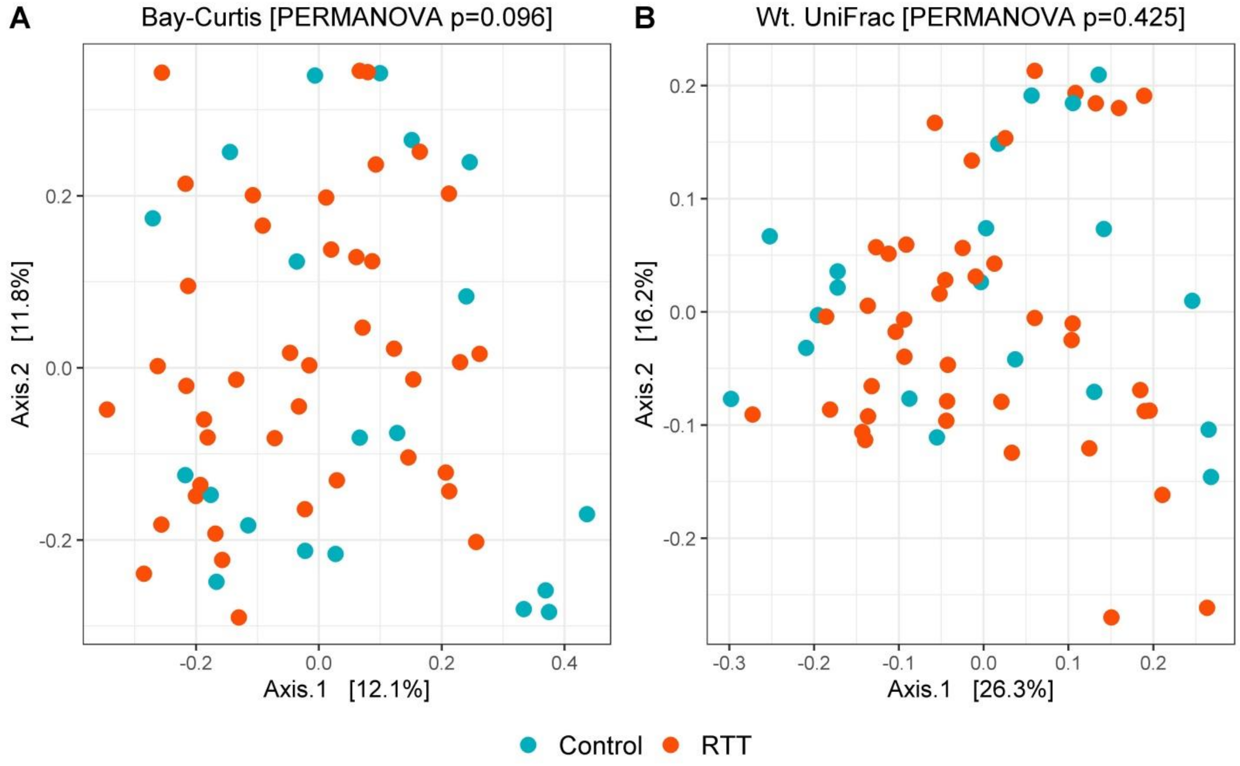

S2 Fig. Principal coordinate analysis (PCoA) plots of Bray-Curtis dissimilarity and weighted UniFrac distance for bacterial communities in individuals with Rett syndrome (RTT) and unaffected controls (C). PERMANOVA analysis using 'Adonis' function in 'vegan' R-package did not reveal clusters of samples between the RTT and C groups (p >0.05). Axis labels represent the percentage of variation explained by each principal coordinate axis.

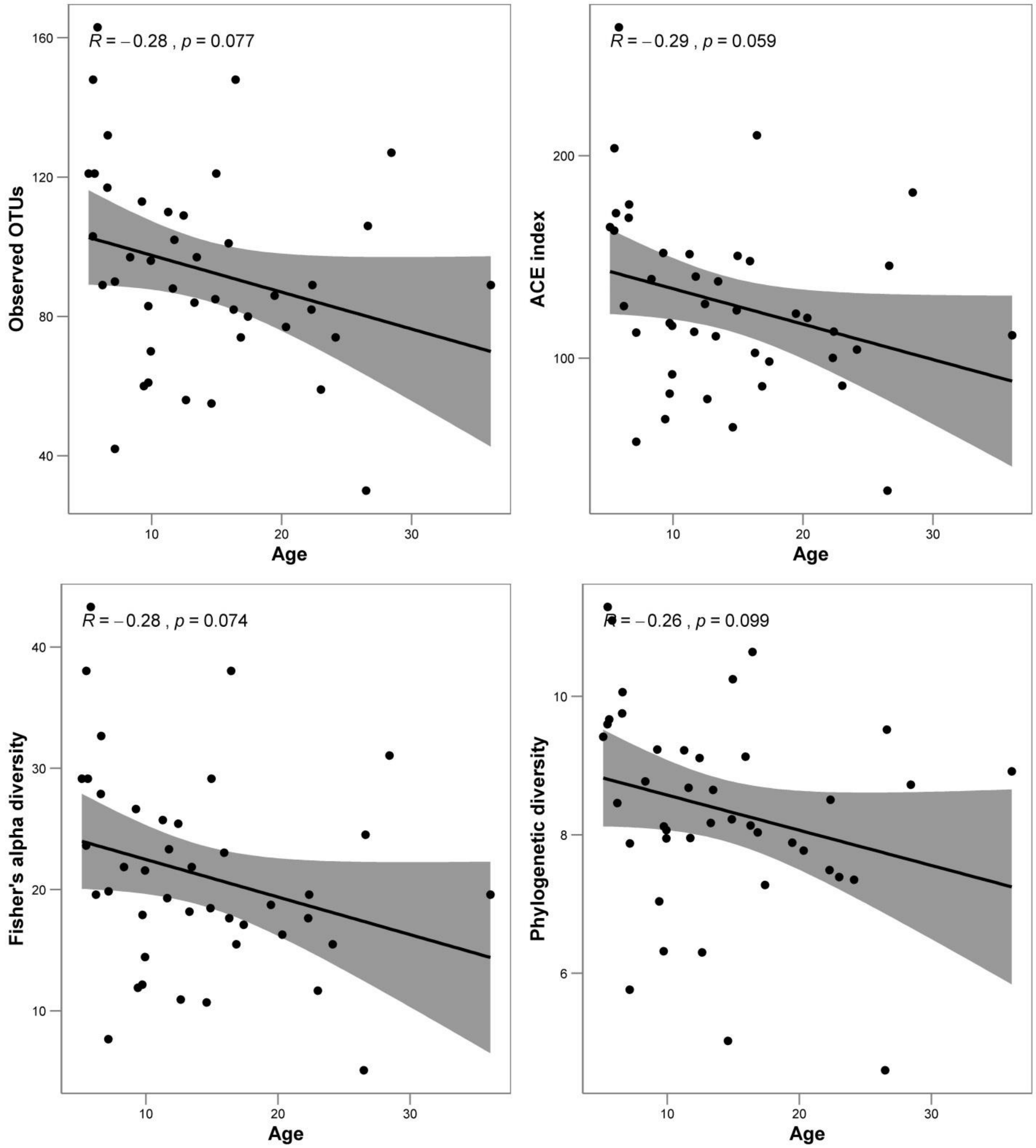

S3 Fig. Correlation between age and alpha diversity of the gut microbiome in individuals with Rett syndrome (RTT). R=Pearson index.

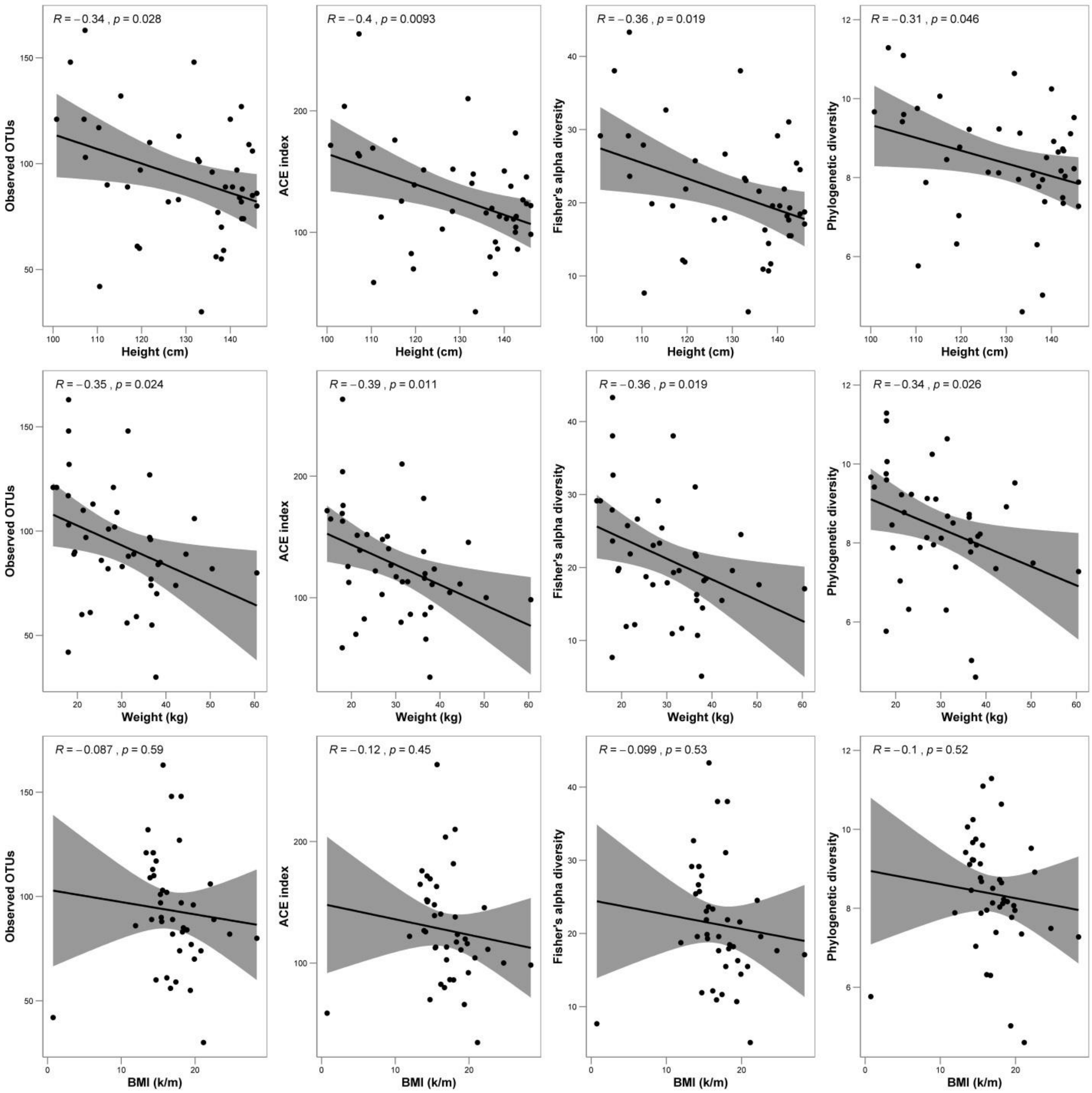

S4 Fig. Correlation between anthropometric measures and alpha diversity of the gut microbiome in individuals with Rett syndrome (RTT).

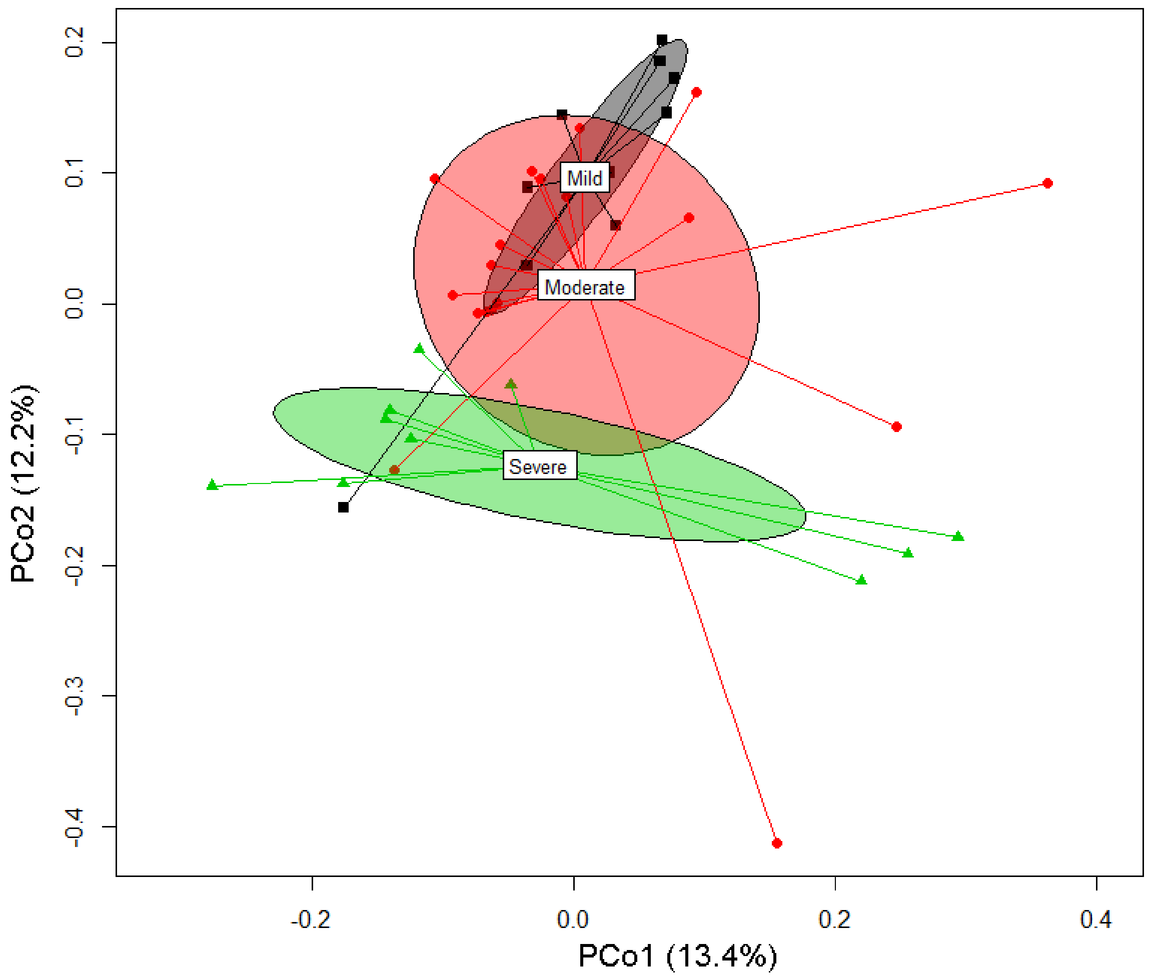

S5 Fig. Dispersion of samples in RTT subjects by clinical severity score (CSS) groups using unweighted UniFrac distance. SD ellipses were generated on the PCoA ordination using *ordispider* and *ordiellipse* functions in *vegan* R-package with type="centroid" at a default confidence interval of 1. The spider connects each sample with the centroid of a particular group. Mild (CSS $\leq$ 19), moderate (CSS = 20-30) and severe (CSS $\geq$ 31) RTT groups are colored as black, red, and green, respectively.

S2 Table. Pairwise PERMANOVA tests of unweighted UniFrac distance matrix in RTT patients by CSS groups.

Variable	Groups	Unweighted UniFrac distance			
		**F-value**	**R^2**	**p-value**	**p-adjust (FDR)**
Clinical severity score (CSS)	mild (n=10) vs moderate (n=18)	0.87	0.03	0.703	0.703
	mild (n=10) vs severe (n=10)	1.91	0.10	0.005*	0.015*
	moderate (n=18) vs severe (n=10)	1.54	0.06	0.02*	0.03*

* Statistically significant

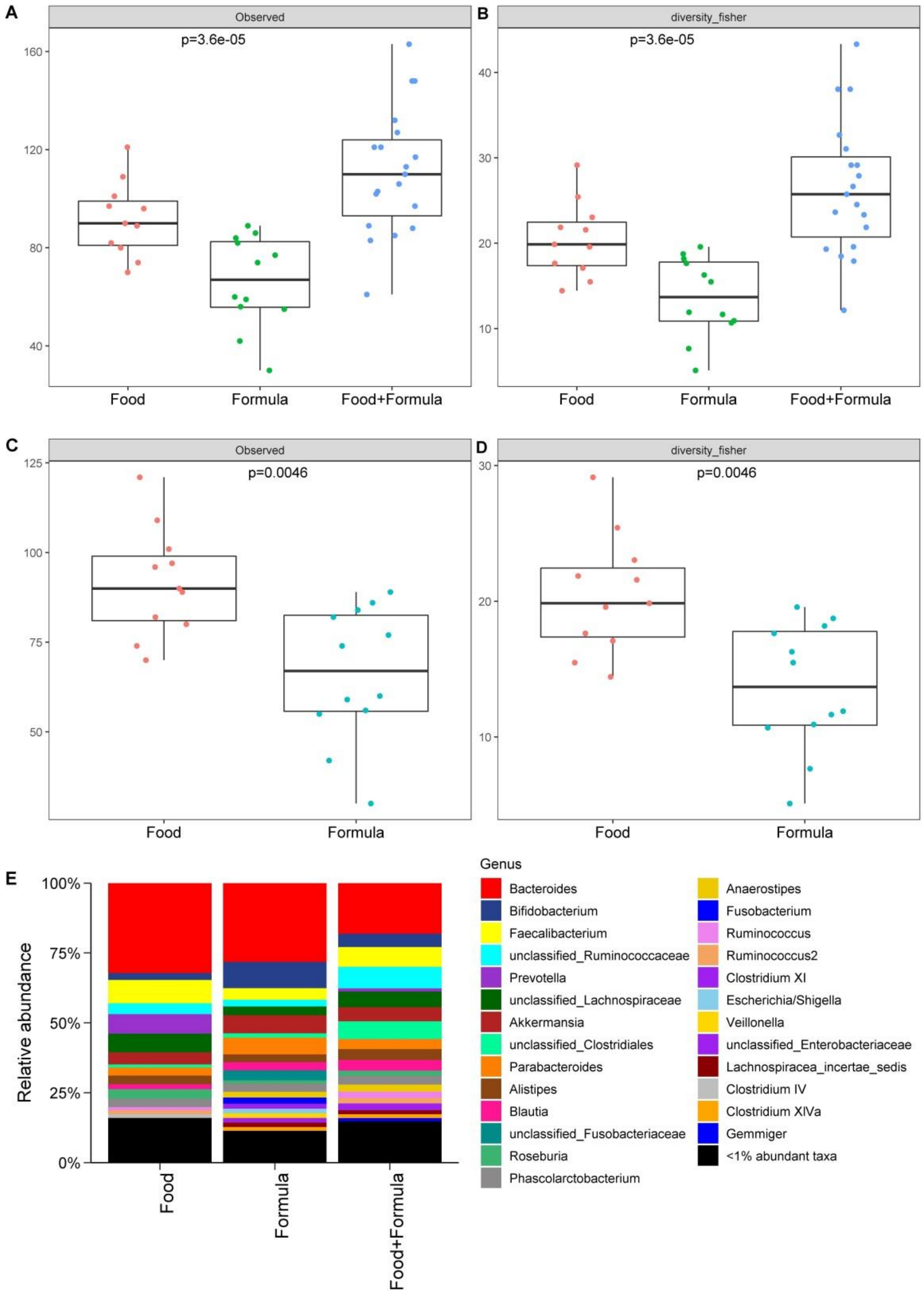

S6 Fig. Gut bacterial diversity and composition in individuals with Rett syndrome (RTT) by dietary source (table food vs formula). The observed OTUs (**A, C**) and Fisher diversity (**B, D**) in the RTT cohort based on consumption of table food or formula; genus level taxa summary of bacterial community in the RTT cohort based on consumption of table food or formula (**E**).

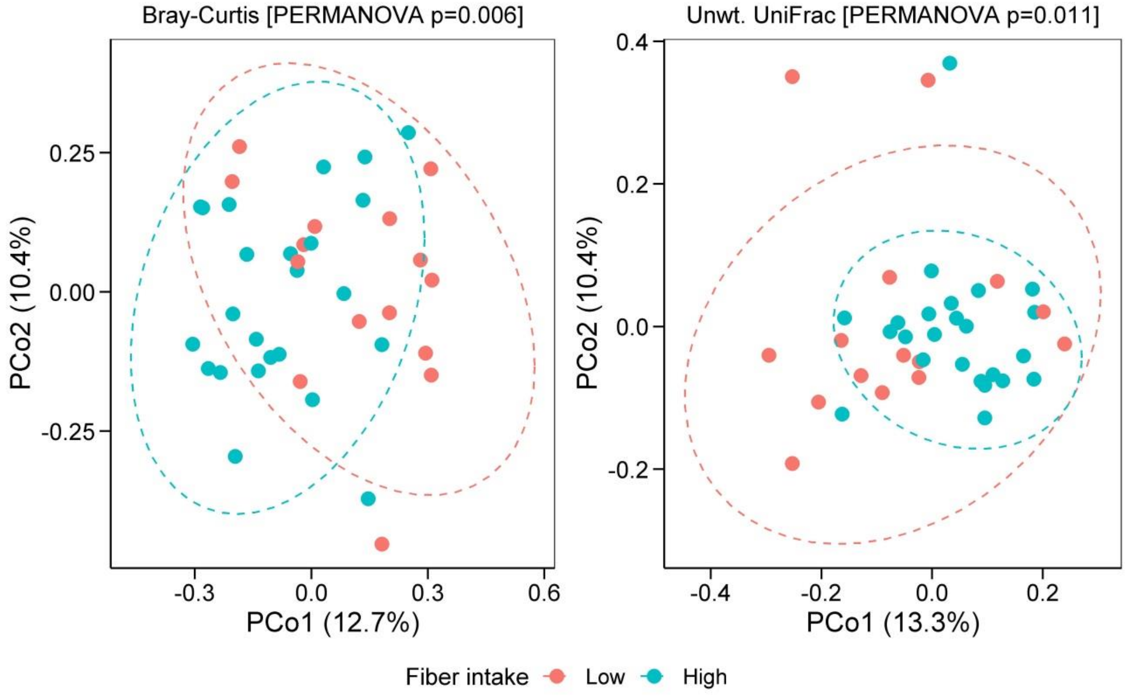

S7 Fig. Gut bacterial composition (beta diversity) in individuals with Rett syndrome (RTT) by dietary fiber source. Low fiber intake=less than 4.1 g fiber daily, High fiber intake=more than 4.1 g fiber daily. Unwt.UniFrac=Unweighted UniFrac distance.

S1 Text. Detailed protocol used for the targeted metabolomics.

Reagents and chemicals: Authentic reference materials for metabolites in the serotonin pathway (including: Tryptophan (Trp), serotonin, melatonin, 5-hydroxyindoleacetic acid (5-HIAA), 5-hydroxytryptophan, and N-acetylserotonin), the dopamine pathway (including tyramine, dopamine, L-DOPA, tyrosine (Tyr), and norepinephrine), and the glutamine cycle (including γ-aminobutyric acid (GABA), glutamine (Gln), glutamate (Glu)), and formic acid (FA), and heptafluorobutyric acid (HFBA) were obtained from Sigma Aldrich (St. Louis, MO, USA). Deuterated analogs of serotonin pathway metabolites (Trp-d5, serotonin-d4, melatonin-d4, and 5-HIAA-d5), dopamine pathway metabolites (tyramine-d4, dopamine-d4, and L-DOPA-d3), and Gln cycle metabolites (GABA-d6, Glu-d5, and Gln-d5) were used as internal standards (IS) and were all obtained from CDN Isotopes (Pointe-Claire, Quebec, Canada). Optimal LC-MS grade water, methanol (MeOH), and acetonitrile (ACN) were obtained from Thermo-Fischer Scientific (Waltham, MA, USA).

Sample preprocessing: Tubes containing 300 gm each of overnight lyophilized stool samples were suspended in water (volume=stool dry weight in mg x 100). The samples were then placed on ice for 30 minutes and centrifuged at 5000*g* for 5 minutes. The collected supernatant samples were sub-aliquotted and stored frozen at -20°C until further use.

Sample preparation: Individual, combined working-IS (WIS) solutions were prepared for the serotonin pathway (Trp-d5, serotonin-d4, melatonin-d4, and 5-HIAA-d5), dopamine pathway (tyramine-d4, dopamine-d4, and L-DOPA-d3), and glutamine cycle (GABA-d6, Glu-d5, and Gln-d5) methods – the concentration of each component in each of the WIS solutions was 500 ng/mL in water. To prepare samples for LC-MS analysis, frozen stool supernatants were thawed and vortex-mixed for 1 minute. The samples were then centrifuged at 10,000*g* for 5 minutes. A

90 µL volume of the appropriate WIS solution was added to a 10 µL volume of stool sample supernatant and vortex-mixed. Samples were transferred into 0.5 mL autosampler vials for analysis.

Liquid Chromatography-Tandem Mass Spectrometry (LC-MS/MS) Hardware and Software: Chromatography was performed on a Shimadzu (Kyoto, Japan) Nexera-XR ultra-high performance liquid chromatography (UHPLC) system consisting of an SIL-30ACMP autosampler, a CTO-20AC column oven, and 2 LC-20ADxr binary pumps. A Sciex 6500 QTRAP mass spectrometer (MS) with a TurboIonSpray® ion emitter installed in a Turbo V™ ionization source was used to acquire tandem MS data using a selected reaction monitoring (SRM) scan mode. The instrument was calibrated using Sciex PPG calibration standard and tuned to the manufacturer's specifications. Chromatographic data were acquired using the Analyst Software® (version 1.6.2) and peak integration, calibration curve regression, and quantification of the unknown samples were performed using SCIEX Multiquant™ Software (version 3.0.1).

Targeted metabolomics method for the serotonin pathway metabolites: Chromatography was performed using a 3-micron Luna C18(2) (150 x 1 mm; 100 Å) analytical and Security Guard C18 (4 x 2 mm) guard column combination from Phenomenex (Torrace, CA, USA). The aqueous mobile phase (A) consisted of H_2O: ACN: FA: HFBA (99.3: 0.5: 0.1: 0.1 v/v/v/v), the organic mobile phase (B) was ACN: FA (99.9: 0.1 v/v), and the needlewash was 1:1 MeOH: water. The chromatographic gradient program consisted of a column temperature of 50°C, an autosampler tray chilling temperature of 10°C, a mobile phase flow rate of 0.100 mL/min, and a gradient elution program specified as follows: 0-0.2 min, 5% B; 0.2-9 min, 5-60% B; 9-9.1 min, 60-5% B; 9.1-12 min, 5% B; with an overall cycle time of 12.4 min per acquisition.

The MS was operated in the positive ion mode under the following global instrument parameters: curtain gas: 20 psi; collision gas: HIGH; ion spray voltage: +5,000 eV; ion source gas 1: 25 psi; ion source gas 2: 25 psi; source temp: 200°C; Q1 and Q3 resolution: unit; and, scan time per transition: 40 ms.

Molecule-specific parameters for the serotonin pathway metabolites included the following de-clustering (DP), entrance (EP), and cell-exit potentials (CXP) for each compound: Trp and Trp-d5: DP: 35 eV, EP: 7 eV, CXP: 15 eV; serotonin and serotonin-d4: DP: 20 eV, EP: 8 eV, CXP: 10 eV; melatonin and melatonin-d4: DP: 45 eV, EP: 6 eV, CXP: 15 eV; 5-HIAA and 5-HIAA-d5: DP: 70 eV, EP: 8 eV, CXP: 17 eV; 5-hydroxytryptophan: DP: 40 eV, EP: 7 eV, CXP: 23 eV; and, N-acetylserotonin: DP: 40 eV, EP: 8 eV, CXP: 10 eV. SRM transitions monitored with collisions energies (CE) were as follows: Trp, 205.1 → 188.1 (15 eV) and 205.1 → 146.1 (25 eV); Trp-d5, 210.1 → 192.1 (15 eV) and 210.1 → 150.1 (25 eV); serotonin, 177.1 → 160.1 (17 eV) and 177.1 → 115.1 (38 eV); serotonin-d4, 181.1 → 164.1 (17 eV) and 181.1 → 119.1 (38 eV); melatonin, 233.1 → 174.1 (22 eV) and 233.1 → 159.1 (39 eV); melatonin-d4, 237.1 → 178.1 (22 eV) and 237.1 → 163.1 (39 eV); 5-HIAA, 192.1 → 146.1 (23 eV) and 192.1 → 118.1 (40 eV); 5-HIAA-d5, 197.1 → 150.1 (23 eV) and 197.1 → 122.1 (40 eV); 5-hydroxytryptophan, 221.1 → 204.1 (16 eV) and 221.1 → 162.1 (26 eV); N-acetylserotonin, 219.1 → 160.1 (23 eV) and 219.1 → 115.1 (49 eV). A 5 µL sample volume was injected on column for analysis.

Targeted metabolomics method for the dopamine pathway metabolites: Chromatography was performed using a 2.7-micron Raptor C18 (100 x 2.1 mm) analytical and Ultra C18 (10 x 2.1 mm) guard column combination from Restek (Bellefonte, PA, USA). The mobile phase and needlewash solutions are the same as describe above for the serotonin method. The chromatographic gradient program consisted of a column temperature of 40°C, an autosampler

tray chilling temperature of 10°C, a mobile phase flow rate of 0.200 mL/min, and a gradient elution program specified as follows: 0-0.50, 5% B; 0.50-6 min, 5-90% B; 6-7 min, 90% B; 7-7.1 min, 90-5% B; 7.1-12 min, 5% B; with an overall cycle time of 12.4 min per acquisition.

The MS was operated in the positive ion mode under the following global instrument parameters: curtain gas: 20 psi; collision gas: HIGH; ion spray voltage: +5,000 eV; ion source gas 1: 25 psi; ion source gas 2: 25 psi; source temp: 200°C; Q1 and Q3 resolution: unit; and, scan time per transition: 50 ms.

Molecule-specific parameters for the dopamine pathway metabolites included the following DPs, EPs, and CXPs for each compound: tyramine and tyramine-d4: DP: 40 eV, EP: 8 eV, CXP: 10 eV; dopamine and dopamine-d4: DP: 40 eV, EP: 9 eV, CXP: 10 eV; L-DOPA and L-DOPA-d3: DP: 50 eV, EP: 7 eV, CXP: 9 eV; Tyr: DP: 50 eV, EP: 8 eV, CXP: 10 eV; and, norepinephrine: DP: 40 eV, EP: 9 eV, CXP: 9 eV. SRM transitions monitored with collisions energies (CE) were as follows: tyramine, 138.1 → 121.1 (16 eV) and 138.1 → 103.1 (29 eV); tyramine-d4, 142.1 → 125.1 (16 eV) and 142.1 → 106.1 (29 eV); dopamine, 154.1 → 137.1 (15 eV) and 154.1 → 91.1 (32 eV); dopamine-d4, 158.1 → 141.1 (15 eV) and 158.1 → 95.1 (32 eV); L-DOPA, 198.1 → 181.1 (14 eV) and 198.1 → 152.1 (20 eV); L-DOPA-d3, 201.1 → 184.1 (14 eV) and 201.1 → 155.1 (20 eV); Tyr, 182.1 → 165.1 (14 eV) and 182.1 → 136.1 (19 eV); and, norepinephrine, 170.1 → 152.1 (12 eV) and 170.1 → 107.1 (28 eV). A 5 µL sample volume was injected on column for analysis.

Targeted metabolomics method for glutamine cycle metabolites: For GABA, Gln, and Glu analysis, the chromatographic system was developed using a 2.6-micron Accucore pentafluorophenyl (PFP) (150 x 2.1 mm) analytical and a 2.6-micron Accucore Defender PFP (10 x 2.1 mm) guard column combination from Thermo Fisher Scientific (Waltham, MA, USA).

The mobile phase and needlewash solutions used were consistent with those described for the serotonin and dopamine methods described above. The chromatographic gradient program consisted of a column temperature of 45°C, an autosampler tray chilling temperature of 5°C, a mobile phase flow rate of 0.300 mL/min, and a gradient elution program specified as follows: 0-0.25 min, 1% B; 0.25-3.0 min, 1-25% B; 3.0-3.1 min, 25-80% B; 3.1-4.0, 80% B; 4.0-4.1 min, 80-1% B; 4.1-7 min, 1% B; with an overall cycle time of 7.4 min per acquisition, and an operational back pressure of ~3,100 psi at initial conditions.

The MS was operated in the positive ion mode under the following global instrument parameters: curtain gas: 25 psi; collision gas: HIGH; ion spray voltage: +4,500 eV; ion source gas 1: 40 psi; ion source gas 2: 50 psi; source temp: 300°C; Q1 and Q3 resolution: unit; and, scan time per transition: 50 ms.

Molecule-specific parameters for the glutamine cycle metabolites included the following DPs, EPs, and CXPs for each compound: GABA and GABA-d6: DP: 80 eV, EP: 9 eV, CXP: 9 eV; Glu, Glu-d5, and Gln: DP: 30 eV, EP: 9 eV, CXP: 11 eV; and, Gln-d5: DP: 40 eV, EP: 9 eV, CXP: 11 eV. SRM transitions monitored with collisions energies (CE) were as follows: GABA, 104.1 → 87.1 (20 eV) and 104.1 → 69.1 (30 eV); GABA-d6, 110.1 → 92.1 (20 eV) and 110.1 → 73.1 (30 eV); Glu, 148.1 → 84.0 (24 eV), 148.1 → 130.1 (14 eV), and 148.1 → 102.1 (17 eV); Glu-d5, 153.1 → 88.1 (24 eV), 153.1 → 135.1 (14 eV), and 153.1 → 107.1 (17 eV); Gln, 147.1 → 130.1 (14 eV) and 147.1 → 84.1 (24 eV); and, Gln-d5, 152.1 → 135.1 (14 eV) and 152.1 → 88.1 (24 eV). A 5 µL sample volume was injected on column for analysis.

Data analysis: We used SCIEX Multiquant™ Software (version 3.0.1) for all our SRM metabolomics data analysis.
